# Association between stress hyperglycemic ratio and stroke in older people with metabolic syndrome: a prospective cohort study from UK Biobank

**DOI:** 10.3389/fendo.2026.1758847

**Published:** 2026-04-13

**Authors:** Chunli Zeng, Hequn Lyu, Lihong Lou, Xixue Lu, Ming Lei

**Affiliations:** 1 Department of Critical Care Medicine, Seventh People’s Hospital of Shanghai University of Traditional Chinese Medicine, Shanghai, China; 2 Department of Lung Disease, Yancheng TCM Hospital Affiliated to Nanjing University of Chinese Medicine, Yancheng, China; 3 Department of Acupuncture and Rehabilitation, Affiliated Hospital of Nanjing University of Chinese Medicine, Nanjing, China; 4 Department of Acupuncture, Yancheng TCM Hospital Affiliated to Nanjing University of Chinese Medicine, Yancheng, China; 5 Department of Acupuncture, Affiliated Hospital of Traditional Chinese Medicine, Shandong First Medical University, Jinan, China

**Keywords:** cohort study, ischemic stroke, stress hyperglycemic ratios, stroke, subgroup analyses

## Abstract

**Background:**

The association between disorders of glucose metabolism and cerebrovascular disease has received increasing attention, but the association between stress hyperglycemic ratios (SHR) and stroke in older people with metabolic syndrome is still unclear; therefore, we used a large-sample cohort study to explore their association.

**Methods:**

60,931 participants aged ≥60 years were included in this cohort. SHR is a new composite indicator that combines fasting blood glucose and glycated hemoglobin. The definition of metabolic syndrome encompasses abdominal obesity, hyperlipidemia, hypertension, and hyperglycemia. The association between SHR and stroke in older people with metabolic syndrome was explored using Cox proportional hazards models. Restricted cubic spline plots were used to explore the presence of nonlinear associations. Inflection points were calculated with recursive methods. KM survival curves were performed to investigate the risk of stroke for different SHR levels over time.

**Results:**

After controlling the confounding of all covariates, we found no significant association between SHR and stroke and its subtypes in older people with metabolic syndrome. Further RCS revealed a nonlinear association only among stroke and ischemic stroke. An inflection point of 0.87 was found, and the association of SHR with stroke and ischemic stroke before and after the inflection point was opposite and both statistically significant. Subgroup analyses did not reveal significant differences.

**Conclusion:**

This prospective cohort study revealed a nonlinear association of SHR with stroke and ischemic stroke in older people with metabolic syndrome, which provides a reference for exploring disorders of glucose metabolism and stroke risk.

## 1 Introduction

Stroke, as a serious cerebrovascular disease, has become one of the leading causes of death and long-term disability worldwide (1, 2). According to the World Health Organization (WHO), stroke occurs in about 15 million people globally every year, and about 6 million of them die as a result, while about 75% of the survivors are left with different degrees of disability, which imposes a heavy economic and mental burden on the patients themselves, their families, and the society (3). Research indicates that the incidence of stroke among individuals with metabolic syndrome is 1.7 times higher than that of the general population (4). Among the many risk factors associated with stroke, hypertension, hyperlipidemia, smoking, alcohol consumption, and obesity are also closely related to metabolic syndrome (5, 6). In recent years, disorders of glucose metabolism have gradually received widespread attention as a key factor in the occurrence and prognosis of stroke (7, 8).

The stress hyperglycemia ratio (SHR) is a new and innovative marker that reflects the degree of control of acute hyperglycemia relative to chronic glucose, which has received much attention in recent years among the research of risk factors for acute and chronic diseases (9, 10). Blood glucose collected at baseline commonly fails to reflect long-term blood glucose levels. Glycosylated hemoglobin A1c (HbA1c) is a well-recognized indicator of blood glucose levels over the past 3 months and can reflect estimated average blood glucose concentrations. Therefore, researchers designed a new metric, SHR, which combines fasting blood glucose and HbA1c and has been shown to more accurately capture levels of stress hyperglycemia (11, 12). Growing evidence suggests that SHR is closely associated with clinical outcomes in patients and has gradually demonstrated considerable potential in evaluating various pathological conditions, including cardiovascular and neurological diseases (13–15). Although existing studies have primarily focused on the prognostic value of SHR in specific patient populations, SHR may also serve as an important biomarker for assessing disease severity in the general population and may exert an independent influence on disease occurrence.

Previous studies of SHR and poor prognosis in cardio-cerebrovascular disease have been extensively explored. SHR has been shown to be independently associated with both short- and long-term mortality in various diseases such as acute coronary syndrome, heart failure, and critical cerebrovascular disease (16–18). Although SHR was initially applied to acute stress scenarios, its essence lies in the relative deviation of current blood glucose levels against the backdrop of long-term glycemic control. Even within relatively stable community-dwelling elderly populations, individual variations in blood glucose responses to physiological stress exist. Consequently, SHR can identify individuals more prone to relative hyperglycemic responses under identical HbA1c conditions, thereby reflecting an underlying predisposition to metabolic stress reactions. Several studies have suggested that higher levels of SHR may reflect greater metabolic stress and inflammatory responses, and thus be involved in the development and evolution of cardio-cerebrovascular events (19–21). However, there is a lack of large-scale prospective population-based data investigating the association between SHR and the risk of primary stroke in older people with metabolic syndrome, and the potential nonlinear relationship and threshold effects between them remain insufficiently investigated. Therefore, based on a British prospective cohort, this study systematically evaluated the association between SHR and the risk of stroke incidence in older people with metabolic syndrome and explored the feasibility of SHR as a stroke biomarker, aiming to provide a new predictive tool and intervention target for the early identification of high-risk groups for stroke.

## 2 Materials and methods

### 2.1 Study population

**Figure 1:**
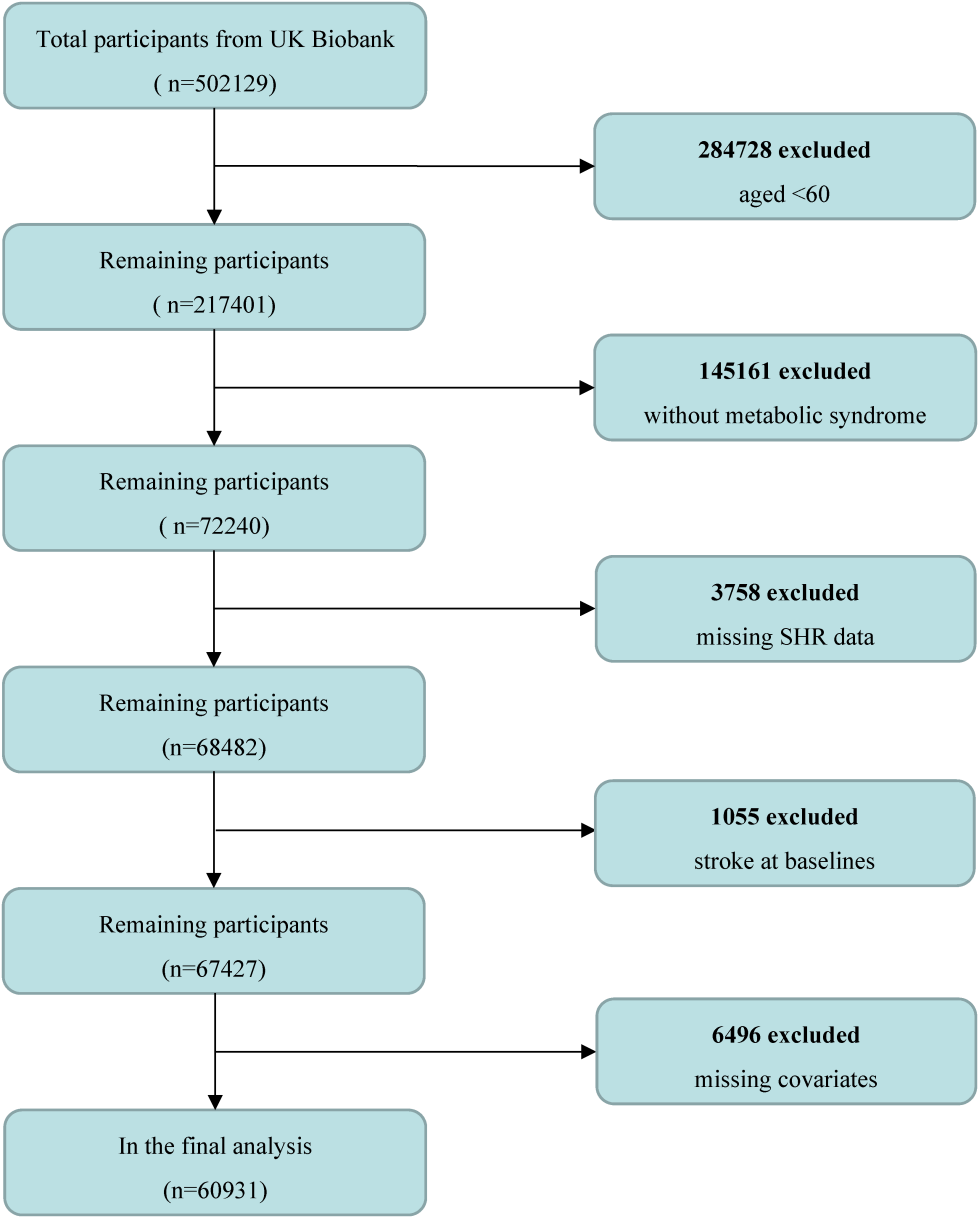
Flow chart of sample selection from UK Biobank.

All participants were drawn from UK Biobank (UKB), a large-scale prospective research program designed to study the impact of lifestyle, environmental, and genetic factors on health and disease by collecting and analyzing large amounts of genetic and phenotypic data. The project recruited >500,000 participants from 2006 to 2010 from the general UK population. Participants completed baseline assessments of demographic characteristics, lifestyle, body measurements, and health status at 22 assessment centers. An approval for the UKB project was obtained from the North West Research Ethics Committee (11/NW/0382) and informed consents were signed by all participants. In this study, the definition of metabolic syndrome was based on the NCEP ATP III-2005 benchmark (22). In this study, we excluded individuals under the age of 60, without metabolic syndrome, missing baseline exposures, those with a history of stroke, and missing data in covariates less than 5% of the study population, ultimately enrolling 60,931 participants (**Figure 1**).

### 2.2 Assessment of stress hyperglycemia ratio

Data for blood glucose(BG) and HbA1c were obtained from blood biochemical tests, with all samples collected under fasting conditions at the same baseline enrollment. The unit of HbA1c in UKB is mmol/mol, which we converted to % according to the formula HbA1c (%) = [HbA1c (mmol/mol)/10.929 + 2.15]. Then we calculate SHR according to the formula [BG (mmol/L)]/[1.59 * HbA1c (%)-2.59] (23).

### 2.3 Assessment of outcomes

In this study, the primary outcome was the incidence of stroke, and secondary outcome were the occurrence of ischemic stroke, intracerebral hemorrhage, and subarachnoid hemorrhage. UKB linked participant data to the National Health Service (NHS) electronic health record in the UK, and information about the participant’s inpatient diagnosis was captured through the hospital’s electronic record, which was typically recorded using International Classification of Diseases (ICD) codes. We combined codes from ICD-9 and ICD-10 for outcome diagnoses. The follow-up period was calculated from the date of enrollment to the date of stroke onset, date of death, date of loss to follow-up, or October 30, 2022, whichever occurred first.

### 2.4 Assessment of covariates

We included a range of covariates to control for the effect of confounding factors on study outcomes. Demographic information included age, gender, ethnicity, and education. Lifestyle included diet score, sleep score, smoking, drinking status, and physical activity. physical measurements were included for BMI, which is strongly associated with stroke. Townsend deprivation index (TDI) and household income were included as economic factors. Hypertension, diabetes, and hyperlipidemia were included as chronic factors for adjustment.

### 2.5 Statistical analysis

We categorized the study population into three groups based on the tertiles of the SHR. Approximately normal continuous variables are expressed as medians ± standard deviations, and non-normally distributed ones as medians (IQR). The following variables with less than 5% missing were excluded: ethnicity, smoking status, drinking status, physical activity, BMI, TDI, and diet score. we interpolated the missing data for household income using the proportional odds logistic regression imputation. To explore the association between SHR and stroke, we developed three Cox proportional hazards models. Model 1 adjusted for gender, age, and ethnicity; model 2 adjusted for physical activity, smoking status, and drinking status added to model 1, and model 3 adjusted for all covariates. In addition, we calculated the HR (95% CI) for the other two tertiles using the second tertile of SHR as reference. To further explore the nonlinear association between SHR and stroke, restricted cubic spline (RCS) were used. After nonlinearity was found, inflection points were calculated using a recursive algorithm, and the association between SHR and stroke before and after the inflection point was calculated with a dichotomous regression model. Kaplan-Meier (KM) survival curves were used to explore the risk of stroke occurrence over time for different SHR classifications. Subgroup analyses and interactions were used to explore whether subgroup differences existed. Finally, we performed sensitivity analyses to verify the stability of the findings. All statistical analyses were performed in R. *P* less than 0.05 was considered statistically significant.

## 3 Results

### 3.1 Baseline characteristics of participants

In this large cohort study from UKB, the mean age of the study population was 64.32 (2.85) years (**Table 1**). The proportion of males and females was 51.75% and 48.25%, respectively. White people, persons comprised 96.77% of the population. The total stroke incidence population was 5,047, with a median follow-up time of 13.45 years. There were 2,396 cases of ischemic stroke, 415 cases of intracerebral hemorrhage and 138 cases of subarachnoid hemorrhage. The study population was grouped by SHR tertiles, those with high SHR were more likely to be white, current drinkers, people with a college degree and household income >51,999. those with SHR at tertile 2 were more likely to have lower risk of diabetes and ischemic stroke.

**Table 1:** Baseline characteristics of participants.

Characteristic	Total	Tertile 1 <0.70	Tertile 2 0.70-0.83	Tertile 3 >0.83	P-value
Age (years)					<0.001
Mean (SD)	64.32 (2.85)	64.40 (2.87)	64.29 (2.84)	64.26 (2.85)	
Gender					<0.001
Female	29,400 (48.25%)	9,777 (48.14%)	10,205 (50.25%)	9,418 (46.37%)	
Male	31,531 (51.75%)	10,534 (51.86%)	10,105 (49.75%)	10,892 (53.63%)	
Ethnicity					<0.001
Non-White	1,969 (3.23%)	994 (4.89%)	495 (2.44%)	480 (2.36%)	
White	58,962 (96.77%)	19,317 (95.11%)	19,815 (97.56%)	19,830 (97.64%)	
Drinking status					<0.001
Never	3,379 (5.55%)	1,334 (6.57%)	1,036 (5.10%)	1,009 (4.97%)	
Previous	2,752 (4.52%)	1,079 (5.31%)	844 (4.16%)	829 (4.08%)	
Current	54,800 (89.94%)	17,898 (88.12%)	18,430 (90.74%)	18,472 (90.95%)	
Smoking status					<0.001
Never	27,542 (45.20%)	1,334 (6.57%)	1,036 (5.10%)	1,009 (4.97%)	
Previous	28,122 (46.15%)	1,079 (5.31%)	844 (4.16%)	829 (4.08%)	
Current	5,267 (8.64%)	17,898 (88.12%)	18,430 (90.74%)	18,472 (90.95%)	
Physical activity					<0.001
No	26,315 (43.19%)	9,016 (44.39%)	8,580 (42.25%)	8,719 (42.93%)	
Yes	34,616 (56.81%)	11,295 (55.61%)	11,730 (57.75%)	11,591 (57.07%)	
Education					<0.001
Unknown	19,051 (31.27%)	6,735 (33.16%)	6,372 (31.37%)	5,944 (29.27%)	
College	13,175 (21.62%)	4,043 (19.91%)	4,332 (21.33%)	4,800 (23.63%)	
Other levels	28,705 (47.11%)	9,533 (46.94%)	9,606 (47.30%)	9,566 (47.10%)	
BMI					<0.001
< 30	32,485 (53.31%)	10,015 (49.31%)	10,853 (53.44%)	11,617 (57.20%)	
≥30	28,446 (46.69%)	10,296 (50.69%)	9,457 (46.56%)	8,693 (42.80%)	
TDI					<0.001
Median (Q1, Q3)	-1.38 (3.01)	-1.24 (3.10)	-1.41 (2.99)	-1.49 (2.94)	
Household income					<0.001
Less than 18,000	24,263 (39.82%)	8,618 (42.43%)	8,012 (39.45%)	7,633 (37.58%)	
18,000 to 30,999	18,933 (31.07%)	6,218 (30.61%)	6,304 (31.04%)	6,411 (31.57%)	
31,000 to 51,999	11,355 (18.64%)	3,545 (17.45%)	3,843 (18.92%)	3,967 (19.53%)	
52,000 to 100,000	5,216 (8.56%)	1,583 (7.79%)	1,750 (8.62%)	1,883 (9.27%)	
Greater than 100,000	1,164 (1.91%)	347 (1.71%)	401 (1.97%)	416 (2.05%)	
Diet score					<0.001
Median (Q1, Q3)	2.78 (1.27)	2.75 (1.29)	2.79 (1.26)	2.80 (1.27)	
Sleep score					<0.001
Median (Q1, Q3)	1.60 (1.04)	1.64 (1.04)	1.58 (1.03)	1.59 (1.04)	
Glucose (mmol/L)					<0.001
Mean (SD)	5.63 (1.72)	4.82 (0.75)	5.30 (0.78)	6.76 (2.37)	
Hba1c					<0.001
Mean (SD)	5.78 (0.81)	5.97 (0.74)	5.64 (0.58)	5.72 (1.00)	
Hypertension					<0.001
No	22,677 (37.22%)	6,959 (34.26%)	7,804 (38.42%)	7,914 (38.97%)	
Yes	38,254 (62.78%)	13,352 (65.74%)	12,506 (61.58%)	12,396 (61.03%)	
Hyperlipidemia					<0.001
No	38,497 (63.18%)	11,940 (58.79%)	13,001 (64.01%)	13,556 (66.75%)	
Yes	22,434 (36.82%)	8,371 (41.21%)	7,309 (35.99%)	6,754 (33.25%)	
Diabetes					<0.001
No	46,132 (75.71%)	14,007 (68.96%)	16,889 (83.16%)	15,236 (75.02%)	
Yes	14,799 (24.29%)	6,304 (31.04%)	3,421 (16.84%)	5,074 (24.98%)	
Stroke					<0.001
No	55,884 (91.72%)	18,492 (91.04%)	18,696 (92.05%)	18,696 (92.05%)	
Yes	5,047 (8.28%)	1,819 (8.96%)	1,614 (7.95%)	1,614 (7.95%)	
Ischemic stroke					0.003
No	58,535 (96.07%)	19,438 (95.70%)	19,567 (96.34%)	19,530 (96.16%)	
Yes	2,396 (3.93%)	873 (4.30%)	743 (3.66%)	780 (3.84%)	
Intracerebral hemorrhage					0.6
No	60,516 (99.32%)	20,164 (99.28%)	20,179 (99.35%)	20,173 (99.33%)	
Yes	415 (0.68%)	147 (0.72%)	131 (0.65%)	137 (0.67%)	
Subarachnoid hemorrhage					0.8
No	60,793 (99.77%)	20,261 (99.75%)	20,265 (99.78%)	20,267 (99.79%)	
Yes	138 (0.23%)	50 (0.25%)	45 (0.22%)	43 (0.21%)	

### 3.2 Association between SHR and stroke in older people with metabolic syndrome

When adjusting for all covariates, we found no significant associations of SHR with stroke[0.96 (0.82, 1.14)] and ischemic stroke[0.99 (0.78, 1.26)] in older people with metabolic syndrome(**Table 2**). Using tertile 2 was used as a reference, a possible U-shaped trend emerged for the association between SHR and stroke with HR (95% CI) of 1.07 (1.00, 1.14) for tertile 1, and 1.02 (0.95, 1.09) for tertile 3. Similar results were observed for the association between SHR and ischemic stroke with HR (95% CI) of 1.11 (1.01, 1.23) for tertile 1 and 1.07 (0.96, 1.18) for tertile 3. However, no significant results were found in the association of SHR with intracerebral hemorrhage and subarachnoid hemorrhage among older people with metabolic syndrome.

**Table 2:** Association between stress hyperglycemic ratio and stroke in older people with metabolic syndrome.

	Model 1 HR (95% CI)	Model 2 HR (95% CI)	Model 3 HR (95% CI)
Stroke			
SHR	0.84 (0.71, 1.00)	0.92 (0.78, 1.09)	0.96 (0.82, 1.14)
SHR category			
Tertile 1	1.12 (1.04, 1.19)	1.08 (1.01, 1.16)	1.07 (1.00, 1.14)
Tertile 2	1.0	1.0	1.0
Tertile 3	1.00 (0.93, 1.07)	1.01 (0.94, 1.08)	1.02 (0.95, 1.09)
Ischemic stroke			
SHR	0.86 (0.68, 1.10)	0.94 (0.74, 1.20)	0.99 (0.78, 1.26)
SHR category			
Tertile 1	1.16 (1.05, 1.28)	1.13 (1.02, 1.24)	1.11 (1.01, 1.23)
Tertile 2	1.0	1.0	1.0
Tertile 3	1.05 (0.95, 1.16)	1.06 (0.96, 1.17)	1.07 (0.96, 1.18)
Intracerebral hemorrhage			
SHR	0.79 (0.44, 1.44)	0.84 (0.46, 1.51)	0.83 (0.46, 1.49)
SHR category			
Tertile 1	1.12 (0.88, 1.42)	1.10 (0.87, 1.39)	1.10 (0.87, 1.39)
Tertile 2	1.0	1.0	1.0
Tertile 3	1.04 (0.82, 1.33)	1.05 (0.83, 1.34)	1.05 (0.82, 1.33)
Subarachnoid hemorrhage			
SHR	0.77 (0.27, 2.22)	0.91 (0.32, 2.58)	0.92 (0.32, 2.60)
SHR category			
Tertile 1	1.15 (0.77, 1.72)	1.09 (0.73, 1.64)	1.09 (0.72, 1.63)
Tertile 2	1.0	1.0	1.0
Tertile 3	0.98 (0.65, 1.49)	1.00 (0.66, 1.52)	1.00 (0.66, 1.52)

Model 1: Age, gender, and ethnicity were adjusted.

Model 2: Age, gender, ethnicity, drinking status, smoking status, and physical activity were adjusted.

Model 3: Age, gender, ethnicity, drinking status, smoking status, physical activity, education, BMI, tdi, household income, score diet, sleep score, diabetes, hypertension, and hyperlipidemia were adjusted.

**Figure 2:**
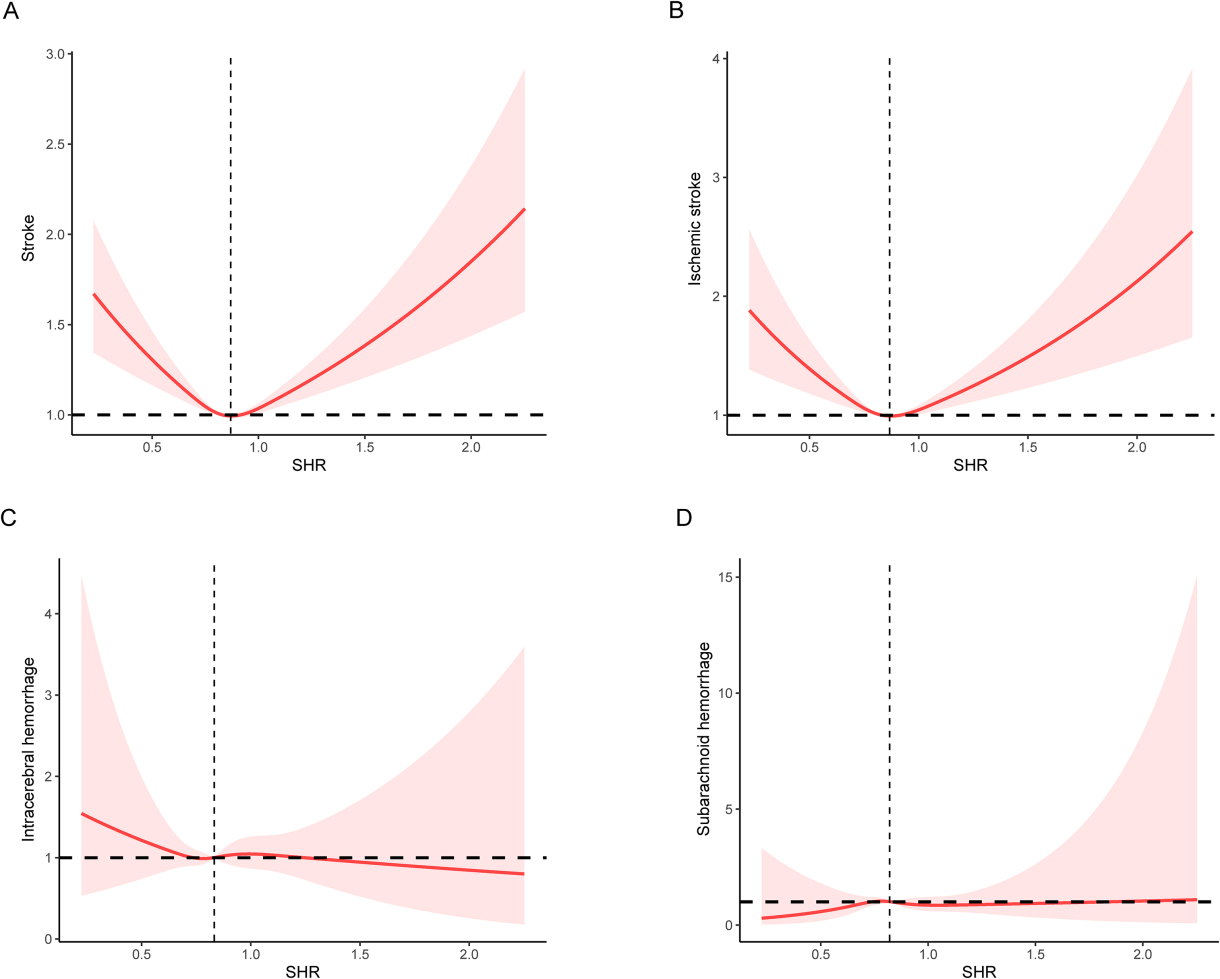
Association between stress hyperglycemic ratio and stroke in older people with metabolic syndrome **(A)** stroke; **(B)** ischemic stroke; **(C)** intracerebral hemorrhage; **(D)** subarachnoid hemorrhage. Age, gender, ethnicity, drinking status, smoking status, physical activity, education, BMI, tdi, household income, score diet, sleep score, hypertension, and hyperlipidemia were adjusted.

**Figure 3:**
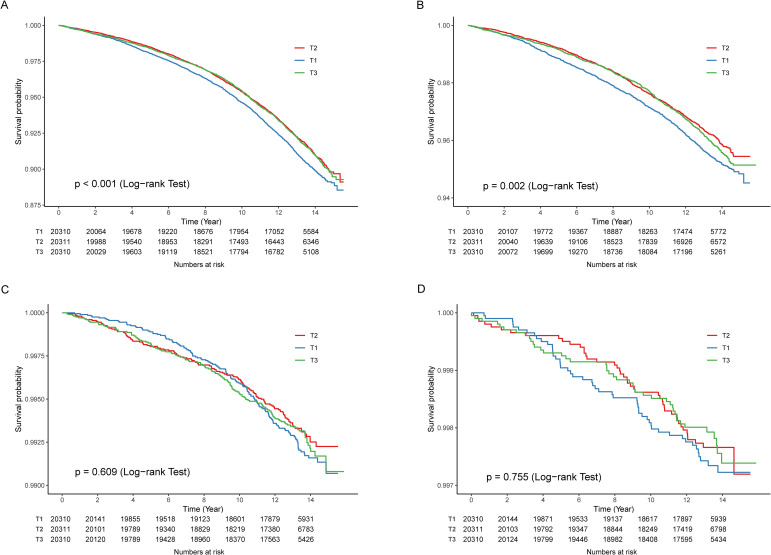
Kaplan–Meier survival analysis **(A)** stroke; **(B)** ischemic stroke; **(C)** intracerebral hemorrhage; **(D)** subarachnoid hemorrhage.

To further explore the dose-effect association between SHR and stroke in older people with metabolic syndrome, we performed the RCS. The results confirmed the U-shaped association of SHR with stroke and ischemic stroke (**Figures 2A, B**). In addition, the results of the KM survival analysis showed that participants at the tertile 1 level had the highest risk of stroke and ischemic stroke (**Figures 3A, B**). After finding the U-shaped relationship, we further calculated the inflection point and the effect of SHR on stroke before and after the inflection point. An inflection point of 0.87 was found, and SHR showed a negative correlation with stroke before the inflection point [0.50 (0.36, 0.69)], and a positive correlation after the inflection point [1.70 (1.36, 2.14)] (**Table 3**). Similar results were found in SHR with ischemic stroke.

**Table 3:** Threshold effect analysis of stress hyperglycemic ratio with stroke and ischemic stroke in older people with metabolic syndrome using a two-piecewise linear regression model.

	Adjust HR (95% CI)	P value
Stroke		
Fitting by standard linear model	0.96 (0.82, 1.14)	0.669
Fitting by two-piecewise linear model		
Inflection point	0.87	
< 0.87	0.50 (0.36, 0.69)	<0.001
> 0.87	1.70 (1.36, 2.14)	<0.001
Log-likelihood ratio	<0.001	
Ischemic stroke		
Fitting by standard linear model	0.99 (0.78, 1.26)	0.947
Fitting by two-piecewise linear model		
Inflection point	0.87	
< 0.87	0.41 (0.26, 0.66)	<0.001
> 0.87	1.96 (1.43, 2.69)	<0.001
Log-likelihood ratio	<0.001	

Age, gender, ethnicity, drinking status, smoking status, physical activity, education, BMI, tdi, household income, score diet, sleep score, diabetes, hypertension, and hyperlipidemia were adjusted.

### 3.3 Subgroup analysis

**Figure 4:**
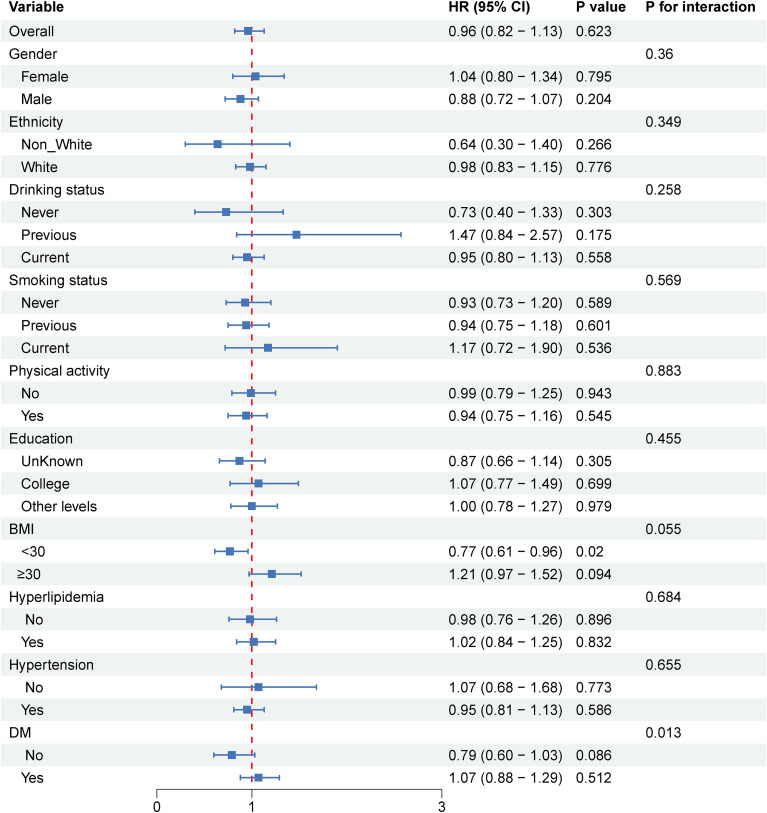
Subgroup analysis of risk factors for the relationship between stress hyperglycemic ratio and stroke in older people with metabolic syndrome.

**Figure 5:**
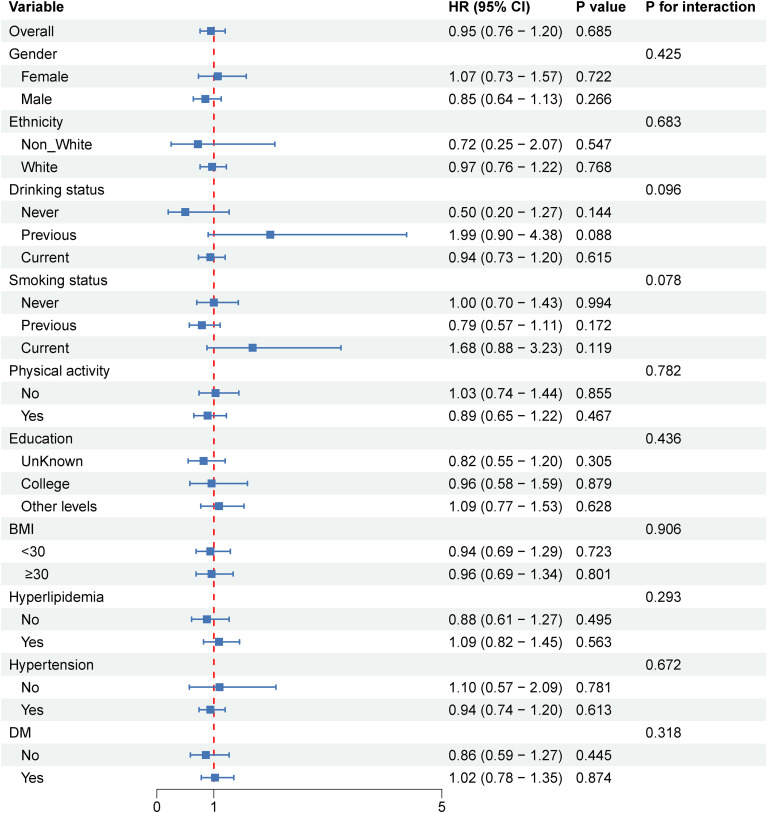
Subgroup analysis of risk factors for the relationship between stress hyperglycemic ratio and ischemic stroke in older people with metabolic syndrome.

To further explore whether there are population-based differences in the association of SHR with stroke and ischemic stroke among older people with metabolic syndrome, we performed subgroup analyses and interactions (**Figures 4**, **5**). In the association between SHR and stroke, no significant differences were found in the subgroups of sex, education, and ethnicity (**Figure 4**). Consistent results were also presented in lifestyle habits (drinking status, smoking status, and physical activity), and no significant interactions emerged in BMI, diabetes, hypertension, and hyperlipidemia. Subgroup analysis of the association between SHR and ischemic stroke also confirmed the stability of the findings (**Figure 5**).

### 3.4 Sensitivity analysis

We performed Cox proportional hazards analyses prior to covariate deletion and imputation. A comparison of the results revealed consistency with the findings presented in **Table 2** (**Supplementary Table 3**). Similarly, the results of the RCS showed no significant differences compared to those after deletion and imputation (**Supplementary Figure S1**).

## 4 Discussion

This prospective cohort study from the UK was the first to explore the association of SHR with stroke and its subtypes. After adjusting for all covariates, the nonlinear associations of SHR with stroke and ischemic stroke were found in older people with metabolic syndrome. Further analyses showed an opposite trend in the association between SHR and stroke before and after the inflection point. The results of the KM survival analyses revealed that people at the first tertile had a highest risk of disease, and the subgroup analyses confirmed the stability of the findings.

In the present study, we observed a significant U-shaped association between SHR and the risk of stroke in older people with metabolic syndrome, and this finding was clinically important and potentially practice-guiding. First, the U-shaped association suggested that whether SHR was too high or too low was associated with an increased risk of stroke. Conventional views tend to emphasize the deleterious role of hyperglycemia in stroke. However, our study further indicated that an abnormally low SHR should not be ignored as well. Lower SHR may reflect inadequate acute glycemic response relative to long-term blood glucose levels, suggesting individuals fail to generate sufficient physiological glucose elevation during stress states. In elderly populations with metabolic syndrome, this may correlate with diminished metabolic reserve, frailty, malnutrition, or abnormal counterregulatory responses—factors that collectively impair the body’s capacity to maintain metabolic homeostasis, thereby increasing susceptibility to cerebrovascular events. Low SHR may aid in identifying high-risk individuals who, despite not exhibiting overt hyperglycemia, harbor underlying metabolic fragility or insufficient physiological reserves. In addition, this U-shaped association provided a new biomarker tool for early identification of stroke risk in older people with metabolic syndrome. Unlike a single glucose value, SHR integrates an individual’s current and basal glycemic status, making it a more dynamic and personalized indicator of metabolic response. Clinicians could incorporate SHR into an early risk stratification system to identify high-risk patient groups and intervene in the pre-stroke phase or high-risk state to prevent stroke before it occurs.

Research on the association between SHR and ischemic stroke among older people with metabolic syndrome has not yet been reported, and most previous studies of SHR and cerebrovascular disease have focused on the general population and adverse outcomes after stroke. A study conducted among middle-aged and elderly individuals in China indicated that elevated SHR was independently associated with an increased risk of stroke, with no evidence of a nonlinear relationship (24). A cohort study from Australia showed that SHR provided good predictive power in predicting adverse outcomes in ischemic stroke (25). Similarly, a study from patients with critical cerebrovascular disease showed SHR to be an independent risk factor for in-hospital mortality in patients, improving the predictive performance of a single model (26). Another study from patients with acute ischemic stroke identified the important potential of SHR for assessing mortality (27). Studies from patients in Intensive Care Unit have shown that SHR is associated with increased short- and long-term mortality in ischemic stroke patients (28). A study from Chinese patients with ischemic stroke after intravenous thrombolysis also confirmed the independent association of SHR with adverse clinical outcomes (29). A perioperative cohort study showed that SHR was associated with an increased risk of hemorrhagic transformation in patients with acute ischemic stroke (30). Studies in patients with spontaneous intracerebral hemorrhage (ICH) have shown that SHR is associated with in-hospital death and cerebral hemorrhage hematoma expansion, and may serve as an adjunct to in-hospital prognosis in cerebral hemorrhage (31). However, our study did not find SHR to be associated with intracerebral hemorrhage.

The mechanism of the association between SHR and stroke remains unclear. The following molecular biological mechanisms may provide some basis for elucidating the association between them. Within a certain range, moderately elevated blood glucose levels can provide brain tissue with more energy material. Brain cells rely mainly on aerobic oxidation of glucose to produce energy under normal physiological conditions and during mild stress (32). When SHR is at a low level but has not yet reached the inflection point, a moderate increase in blood glucose can satisfy the energy needs of brain cells (33), which helps to maintain the normal function and structure of neural cells, and provides a certain degree of protection to brain tissues, thus reducing the risk of stroke. A moderate increase in blood glucose may activate the antioxidant defense system in the body, induce the expression of antioxidant enzymes, enhance the body’s antioxidant capacity, and reduce oxidative stress damage (34). Low SHR may reflect impaired glucose regulation during stress states. Under normal conditions, chronic stress responses promote gluconeogenesis by activating the sympathetic nervous system and the hypothalamic-pituitary-adrenal axis, thereby supplying energy to vital organs (35). However, when this counterregulatory response is compromised, the body may fail to generate sufficient stress-induced hyperglycemia, leading to exacerbated oxidative stress and vascular damage. When SHR exceeds the inflection point, relative hyperglycemia may exacerbate oxidative stress, leading to excessive production of reactive oxygen species (ROS) and reactive nitrogen species (RNS). This exceeds the clearance capacity of the body’s antioxidant system, resulting in an imbalance of oxidative stress (36, 37). Excessive ROS and RNS can damage intracellular biomolecules, disrupt normal cellular structure and function, and impair cerebrovascular endothelial cells. This endothelial dysfunction increases the risk of thrombosis, thereby facilitating the development of stroke. Furthermore, as oxidative stress intensifies, inflammatory responses are activated. Relative hyperglycemia consistently induces the overexpression of inflammatory factors, leading to the release of increased inflammatory mediators and triggering an inflammatory cascade reaction (38). The inflammatory response can lead to inflammatory damage of the blood vessel wall, increase the fragility and permeability of blood vessels, induce or aggravate cerebrovascular lesions, and raise the risk of stroke (39). Relative hyperglycemia can also cause changes in blood rheology, increasing blood viscosity and blood flow resistance, leading to inadequate cerebral blood perfusion (40). At the same time, relative hyperglycemia also activates the coagulation system, promotes platelet aggregation and activation, increases the level of fibrinogen in the blood, and keeps the blood in a hypercoagulable state (41–43). All these factors work jointly to trigger thrombosis and blockage of cerebral blood vessels, leading to the occurrence of ischemic stroke.

Notably, this study did not observe a significant association between SHR and the risk of intracerebral hemorrhage or subarachnoid hemorrhage. Unlike ischemic stroke, hemorrhagic stroke is typically closely associated with structural alterations in the vascular wall. Its primary pathophysiological basis includes long-term hypertension-induced small artery hyalinosis, microaneurysm formation, vascular wall degenerative changes, and aneurysm rupture (44, 45). These processes reflect the cumulative effects of long-term hemodynamic stress and vascular structural damage, with potentially limited associations to short-term metabolic states or relatively elevated blood glucose levels. Therefore, the lack of significant association between SHR and hemorrhagic stroke subtypes in this study partially supports the notion that SHR may primarily influence ischemic stroke risk by affecting thrombosis-related pathways or endothelial dysfunction, rather than through structural rupture pathways of the vascular wall. Furthermore, the differential association patterns observed across stroke subtypes in this study suggest that SHR may function more as an indicator of metabolic stress and vascular dysfunction rather than a non-specific risk marker universally applicable to all types of cerebrovascular events. These findings provide novel insights into the potential mechanisms underlying the role of relative hyperglycemia across different stroke subtypes, although the specific biological mechanisms warrant further investigation and validation.

The following advantages exist in this study. First, UKB is one of the largest cohort studies at present, and the adequate sample size and sufficiently long follow-up time guarantee the credibility of the findings. Secondly, this study not only explored the association between SHR and stroke, but also further explored the association with stroke subtypes, confirming the effect of SHR as a risk marker for different stroke types. Finally, we performed interaction tests to determine the stability of the findings. There are also some limitations of this study that need to be clarified. First, due to the nature of the study, the causal association between exposure and outcomes could not be determined. Second, although sufficient covariates were included to be adjusted to minimize the effects of confounding, the influence of unknown factors on the findings could not be avoided. Third, the association between SHR and stroke may change over time, but limitations of the data prevented examining the impact of longitudinal changes in these factors on outcomes. Future studies should utilize repeated measures data for research and analysis. Fourth, the participants of this study was mostly middle-aged and elderly people in the UK, and the applicability of its findings to other populations needs to be further explored.

## 5 Conclusion

This prospective cohort study revealed a nonlinear relationship of SHR with stroke and ischemic stroke in older people with metabolic syndrome, and both excessively high and low SHR may increase stroke risk. This provides a new perspective to explore the association between glucose metabolism and stroke, and a new tool for screening in high-risk populations. More studies are needed in the future to explore the molecular biological mechanisms underlying the association.

## Data Availability

The original contributions presented in the study are included in the article/**Supplementary Material**. Further inquiries can be directed to the corresponding author.
