# Supplementary material for: Association between stress hyperglycemic ratio and stroke in older people with metabolic syndrome: a prospective cohort study from UK Biobank
Source: Front Endocrinol (Lausanne). 2026 Apr 13;17:1758847. doi: 10.3389/fendo.2026.1758847 (PMC13111165; doi:10.3389/fendo.2026.1758847)

**1.The details of measurement of metabolic syndrome components in UK Biobank.**

HDL (data field:30760), triglycerides (data field:30870), glucose (data field:30740), Waist circumference (data field:48), systolic blood pressure (data field:4080), diastolic blood pressure (data field:4079).

Antihypertensive drugs (data field:20003):

1140888578,1140860470,1140860558,1140860478,1140910606,1141164148,1141164154,1140860882,1140860892,1140860728,1140881706,1140860736,1140860750,1140860758,1140860764,1141150560,1140881714,1140851692,1140888552,1140860776,140860784,1140860790,1140881712,1140860696,1140864952,1140860706,1140864910,1140860714,1140864618,1140888560,1140860802,1141180592,1140860806,1141165470,1141165476,1141188408,1141199940,1140888556,1140860878,1140864176,1140860904,1140860912,1140923712,1141145658,1140866758,1141171336,1141171344,1141156836,1141156846,1141152998,1141153006,1141172682,1141172686,1140916356,1140916362,1141151018,1141179974,1141151016,1141145660,1141145668,1141201038,1141201040,1141193282,1141193346,1141166006,1141187790,1141172492,1141187788,1140872568,1140872472,1141153026,1141153032,1140879802,1140861202,1141200400,1140888646,1141190160,1141187094,1141199858,1141188836,1141188576,1141188152,1141188920,1141200782,1140868036,1141201814,1140928212,1141150500,1140861088,1140860358,1140926188,1140923572,1140861090,1140881702,1140861106,1140861120,1140860426,1140860356,1141173766,1141145870,1141157140,1141150538,1141169730,1140927940,1140861276,1140861282,1140879806,1140861128,1140861136,1140861138,1140926780,1140861166,1140926778,1140917428,1141175224,1141157136,1140911698,1141151474,1141174684,1141167832,1140888510,1141169710,1141150926,1141187774,1140881692,1141153328,1141153316,1140866466,1140866460,1140866554,1140851730,1140860338,1140879810,1140861176,1140861190,1140881894,1140866092,1140866094,1140866090,1140909708,1140866116,1140866412,1140866506,1141195258,1140866332,1140866194,1140851414,1140866406,1140866408,1140866418,1140866248,1140888496,1140864874,1140866122,1140910442,1140860340,1140866450,1141146126,1141194794,1141194800,1140860312,1141194808,1141194810,1140866136,1140866446,1140866162,1140888686,1140866156,1140866158,1140909706,1140866144,1140866410,1140866146,1141180778,1140851484,1140866108,1140866110,1140866078,1141180592,1141180598,1141201244,1141201250,1141146378,1140888922,1140888512,1140866352,1140923276,1140866422,1140866426,1140866416,1140866420,1140927174,1140866226,1140866280,1140866448,1140866282,1140866438,1140866356,1140866388,1140866328,1140866402,1140866236,1140866244,1140866312,1140866318,1140923282,1140866396,1140916342,1140866692,1140879854,1140860304,1140860362,1140860332,1140860292,1140860294,1140910614,1141164276,1141164280,1140860192,1141194804,1140860194,1140875808,1140860278,1140909368,1141168498,1140866724,1140866726,1140866738,1140866756,1140922930,1140860348,1141146124,1140923336,1140860324,1140860328,1141146128,1140879760,1140860434,1140860492,1140864950,1141171152,1141182904,1140879762,1140860498,1140879818,1140860308,1140860404,1140860266,1140860274,1141182968,1140879824,1140860250,1140879830,1140860334,1140860220,1140860222,1140879842,1141156754,1140866704,1140860418,1140866712,1140866764,1140866804,1140866800,1140851556,1140866782,1140866802,1141152076,1140866766,1141187048,1140860336,1140879778,1140860690,1141194372

Antihyperlipidemic drugs (data field:20003):

1140861924,1141157260,1140861926,1140861928,1141201306,1140861954,1140862026,1140862028, 1140861944,1140861856,1141157262,1140861858,1140910670,1141188546,1140861868,1141181868

1140861958,1140881748,1141200040,1141188146,1140888594,1140864592,1140888648,1140861970,1141146234,1141146138,1141192410,1141192414,1140910632,1140910654,1141192736,1141192740,1140861936,1140865576,1141157416,1140861942,1140909780,1141180734,1141180722,1140888590, 1140861848

Antihyperglycemic drugs (data field: 20003):

1140883066,1140884600,1141189090,1141189094,1140874686,1140874744,1140874746,1141157284,1140874652,1140874658,1140874664,

1141152590,1141156984,1140874718,1140874674,1140874706,1140874716,1141168660,1141168668,1141173882,1141173786,1141171646,

1141171652,1141177600,1141177606,1140874826,1140868902,1140868908

| **Characteristic** | **Total** | **Tertile 1**  **<0.70** | **Tertile 2**  **0.70-0.83** | **Tertile 3**  **>0.83** | **P-value** |
| --- | --- | --- | --- | --- | --- |
| **Waist circumference** |  |  |  |  | <0.001 |
| Mean (SD) | 99.71 (11.77) | 100.80 (11.76) | 99.43 (11.36) | 98.89 (12.10) |  |
| **HDL cholesterol** |  |  |  |  | <0.001 |
| Mean (SD) | 1.25 (0.31) | 1.22 (0.28) | 1.26 (0.30) | 1.28 (0.33) |  |
| **Triglycerides** |  |  |  |  | 0.7 |
| Mean (SD) | 2.34 (1.07) | 2.35 (1.06) | 2.34 (1.03) | 2.34 (1.11) |  |
| **Systolic blood pressure** |  |  |  |  | <0.001 |
| Mean (SD) | 146.42 (17.54) | 145.15 (17.37) | 146.70 (17.37) | 147.41 (17.80) |  |
| **Diastolic blood pressure** |  |  |  |  | <0.001 |
| Mean (SD) | 83.66 (9.71) | 83.32 (9.73) | 84.29 (9.56) | 83.36 (9.82) |  |
| **Antihypertensive drugs** |  |  |  |  | <0.001 |
| No | 29,041 (47.66%) | 9,043 (44.52%) | 10,014 (49.31%) | 9,984 (49.16%) |  |
| Yes | 31,890 (52.34%) | 11,268 (55.48%) | 10,296 (50.69%) | 10,326 (50.84%) |  |
| **Antihyperlipidemic drugs** |  |  |  |  | <0.001 |
| No | 30,911 (50.73%) | 8,788 (43.27%) | 10,485 (51.62%) | 11,638 (57.30%) |  |
| Yes | 30,020 (49.27%) | 11,523 (56.73%) | 9,825 (48.38%) | 8,672 (42.70%) |  |
| **Antihyperglycemic drugs** |  |  |  |  | <0.001 |
| No | 53,870 (88.41%) | 17,322 (85.28%) | 19,080 (93.94%) | 17,468 (86.01%) |  |
| Yes | 7,061 (11.59%) | 2,989 (14.72%) | 1,230 (6.06%) | 2,842 (13.99%) |  |

**2.** International Classification of Diseases (ICD) codes of diagnosing stroke

| Outcome | ICD-9 | ICD-10 |
| --- | --- | --- |
| Stroke | 4309,4319,4340,4341,4349,4369 | I60,I61,I62,I63, I64,I67,I69 |
| Ischemic stroke | 4340,4341,4349,4369 | I63, I64 |
| Intracerebral hemorrhage | 4319 | I61 |
| Subarachnoid hemorrhage | 4309 | I60 |

**3.** Sensitivity analysis of association between stress hyperglycemic ratio and stroke

|  | **Model 1 HR (95% CI)** | **Model 2 HR (95% CI)** | **Model 3 HR (95% CI)** |
| --- | --- | --- | --- |
| **Stroke** |  |  |  |
| SHR | 0.87 (0.73, 1.05) | 0.95 (0.79, 1.15) | 1.00 (0.83, 1.20) |
| SHR category |  |  |  |
| Tertile 1 | 1.11 (1.03, 1.20) | 1.08 (1.00, 1.16) | 1.08 (1.01, 1.15) |
| Tertile 2 | 1.0 | 1.0 | 1.0 |
| Tertile 3 | 1.01 (0.94, 1.09) | 1.03 (0.95, 1.11) | 1.03 (0.96, 1.12) |
| **Ischemic stroke** |  |  |  |
| SHR | 0.95 (0.73, 1.24) | 1.04 (0.80, 1.35) | 1.09 (0.84, 1.42) |
| SHR category |  |  |  |
| Tertile 1 | 1.14 (1.02, 1.27) | 1.10 (0.99, 1.23) | 1.10 (1.01, 1.21) |
| Tertile 2 | 1.0 | 1.0 | 1.0 |
| Tertile 3 | 1.08 (0.96, 1.20) | 1.09 (0.97, 1.21) | 1.10 (0.98, 1.22) |
| **Intracerebral hemorrhage** |  |  |  |
| SHR | 0.90 (0.48, 1.71) | 0.94 (0.50, 1.78) | 0.93 (0.49, 1.76) |
| SHR category |  |  |  |
| Tertile 1 | 1.10 (0.85, 1.43) | 1.09 (0.84, 1.41) | 1.09 (0.84, 1.41) |
| Tertile 2 | 1.0 | 1.0 | 1.0 |
| Tertile 3 | 1.06 (0.82, 1.38) | 1.07 (0.82, 1.39) | 1.07 (0.82, 1.38) |
| **Subarachnoid hemorrhage** |  |  |  |
| SHR | 0.76 (0.24, 2.45) | 0.89 (0.28, 2.83) | 0.92 (0.29, 2.92) |
| SHR category |  |  |  |
| Tertile 1 | 1.12 (0.71, 1.75) | 1.07 (0.68, 1.68) | 1.06 (0.67, 1.66) |
| Tertile 2 | 1.0 | 1.0 | 1.0 |
| Tertile 3 | 0.97 (0.61, 1.54) | 0.99 (0.62, 1.57) | 0.99 (0.62, 1.57) |

Model 1: Age, gender, and ethnicity were adjusted.

Model 2: Age, gender, ethnicity, drinking status, smoking status, and physical activity were adjusted.

Model 3: Age, gender, ethnicity, drinking status, smoking status, physical activity, education, BMI, tdi, household income, score diet, sleep score, diabetes, hypertension, and hyperlipidemia were adjusted.

**Figure S1.** Sensitivity analysis of association between stress hyperglycemic ratio and stroke in older people with metabolic syndrome (A: stroke; B: ischemic stroke; C: intracerebral hemorrhage; D: intracerebral hemorrhage).


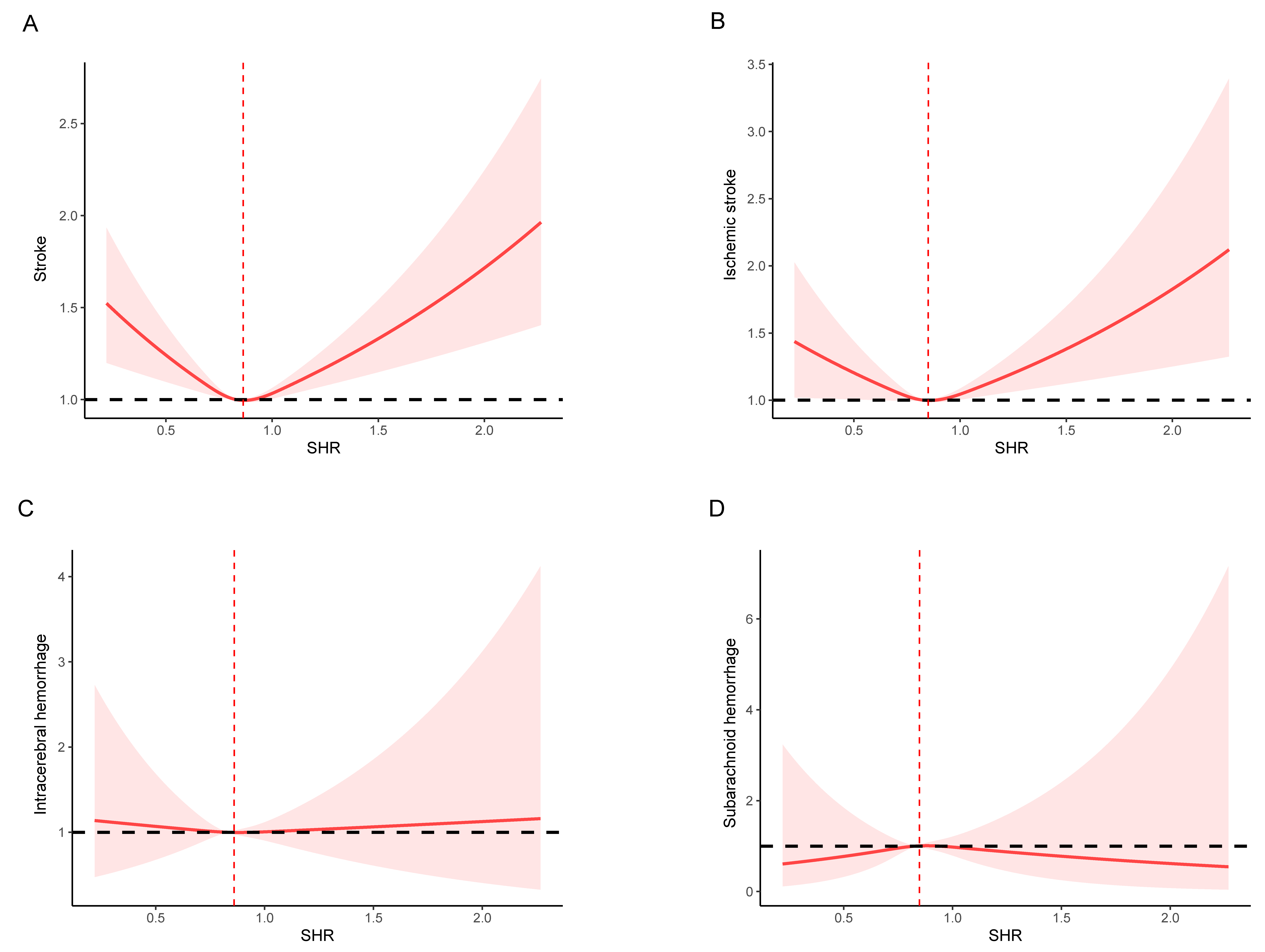

Supplement: Supplementary file 1 [file DataSheet1.docx]
